# The Influence of Different Knowledge-Driven Methods on Landslide Susceptibility Mapping: A Case Study in the Changbai Mountain Area, Northeast China

**DOI:** 10.3390/e21040372

**Published:** 2019-04-05

**Authors:** Zhongjun Ma, Shengwu Qin, Chen Cao, Jiangfeng Lv, Guangjie Li, Shuangshuang Qiao, Xiuyu Hu

**Affiliations:** College of Construction Engineering, Jilin University, Changchun 130026, China

**Keywords:** landslide susceptibility mapping, Changbai Mountain area, rough set, AHP, entropy weight method, Cohen’s kappa index, GIS

## Abstract

Landslides are one of the most frequent geomorphic hazards, and they often result in the loss of property and human life in the Changbai Mountain area (CMA), Northeast China. The objective of this study was to produce and compare landslide susceptibility maps for the CMA using an information content model (ICM) with three knowledge-driven methods (the artificial hierarchy process with the ICM (AHP-ICM), the entropy weight method with the ICM (EWM-ICM), and the rough set with the ICM (RS-ICM)) and to explore the influence of different knowledge-driven methods for a series of parameters on the accuracy of landslide susceptibility mapping (LSM). In this research, the landslide inventory data (145 landslides) were randomly divided into a training dataset: 70% (81 landslides) were used for training the models and 30% (35 landslides) were used for validation. In addition, 13 layers of landslide conditioning factors, namely, altitude, slope gradient, slope aspect, lithology, distance to faults, distance to roads, distance to rivers, annual precipitation, land type, normalized difference vegetation index (NDVI), topographic wetness index (TWI), plan curvature, and profile curvature, were taken as independent, causal predictors. Landslide susceptibility maps were developed using the ICM, RS-ICM, AHP-ICM, and EWM-ICM, in which weights were assigned to every conditioning factor. The resultant susceptibility was validated using the area under the ROC curve (AUC) method. The success accuracies of the landslide susceptibility maps produced by the ICM, RS-ICM, AHP-ICM, and EWM-ICM methods were 0.931, 0.939, 0.912, and 0.883, respectively, with prediction accuracy rates of 0.926, 0.927, 0.917, and 0.878 for the ICM, RS-ICM, AHP-ICM, and EWM-ICM, respectively. Hence, it can be concluded that the four models used in this study gave close results, with the RS-ICM exhibiting the best performance in landslide susceptibility mapping.

## 1. Introduction

Landslides are one of the most frequent geomorphic hazards, and they have considerable economic and ecological consequences [1,2,3]. To mitigate these social and economic losses, it is valuable and essential to assess the landslide susceptibility in a region. Therefore, in recent years, the assessment of landslide susceptibility, which refers to the likelihood of a landslide occurring in an area on the basis of the local terrain and environmental conditions, has become a topic of major interest [4,5]. Landslide susceptibility mapping (LSM) is considered in the decision-making process involving land use management as an efficient approach to reduce property damage and economic loss in landslide-prone areas [1,6,7,8,9]. The outcome maps would be useful for general planned development activities and disaster management in the future, such as choosing new urban areas and infrastructural activities, as well as for environmental protection.

Landslide susceptibility maps can be obtained using both qualitative (inventory-based and knowledge-driven methods) or quantitative approaches (data-driven methods and physically based models) [4,10,11,12,13,14,15,16,17].

Landslide inventory-based techniques, as a prelude to all other methods, include the collection of past landslide data, construction of databases, and production of susceptibility maps based on those data [18]. Landslide inventory mapping can be carried out using a variety of methods that were updated and summarized by Corominas et al. [17].

Knowledge-driven methods that estimate landslide potential from the practical experience and expertise of the researcher are used by geomorphologists to analyze aerial photographs or to conduct field surveys [19].

Data-driven landslide susceptibility assessment methods are used to select and analyze factors affecting landslides in areas with environmental conditions similar to those where past landslides have been reported [17]. They can be grouped in bivariate statistical analysis, multivariate statistical analysis and active learning statistical analysis. In bivariate statistical analysis, the weights of the landslide conditioning factors are assigned based on landslide density using different methods—including frequency ratio (FR) [13,15,20,21,22], the information content model (ICM) [23,24], weight of evidence (WoE) [16], certainty factors (CF) [25], favorability functions (FF) [26], and the likelihood ratio model (LRM) [27]. The multivariate statistical methods evaluate the combined relationship between a dependent variable (landslide occurrence) and a series of independent variables (landslide controlling factors), and the most popular methods to analyze the resulting matrix include logistic regression (LR) [6,13,28,29,30,31,32,33], discriminant analysis (DA) [34,35], random forest (RF) [36,37,38] and active learning statistical analysis, such as the artificial neural networks (ANNs) [3,6,39,40,41,42].

Physically based methods, such as deterministic techniques, are based on mathematical modeling of the physical mechanisms controlling slope failure [43,44,45,46,47,48,49]. However, it is reported that the methods are only applicable over large areas when the geological and geomorphological conditions are fairly homogeneous and the landslide types are simple [17].

Moreover, several studies have used two or more models to produce landslide susceptibility maps and compare their accuracy [3,15,16,36,50,51,52,53].

Although many approaches are available for producing landslide susceptibility maps, the focus in this paper is on the influence of different knowledge-driven methods for a series of parameters on the accuracy of LSM. A comparative study between the ICM using three knowledge-driven methods, that is, rough set (RS), artificial hierarchy process (AHP), and entropy weight method (EWM), has been considered so far in the literature. Therefore, four models, namely, ICM, AHP-ICM, EWM-ICM, and RS-ICM, were used to produce landslide susceptibility maps in the Changbai Mountain area (CMA). The four models were validated based on receiver operating characteristic (ROC) curves and the Cohen’s kappa index, and the influence of different knowledge-driven methods is discussed in the study.

## 2. Study Area

**Figure 1:**
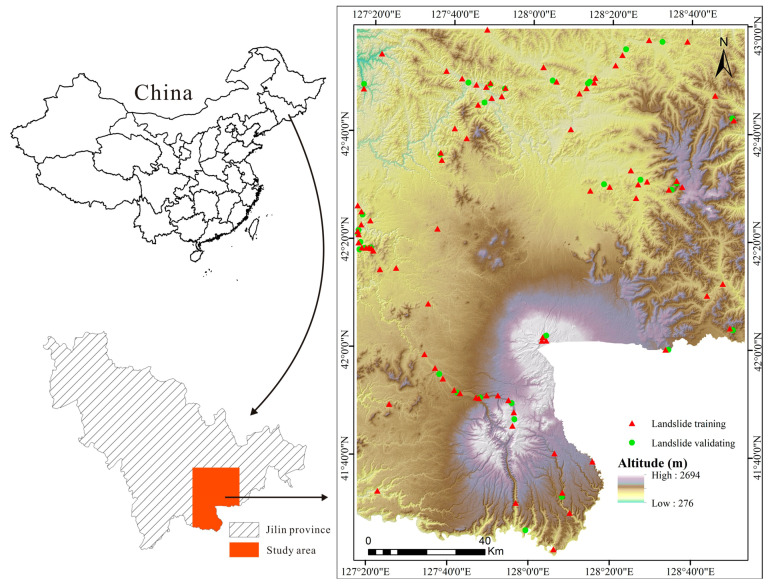
Study area and shaded relief image showing the surface.

The CMA is located in northeastern China and is bounded by North Korea (Figure 1) [54], between approximately 41°21′N and 43°01′N and 127°01′E and 128°54′E, where the climate is temperate continental monsoon with mean annual temperatures of −7.3 to +4.8 °C and average annual precipitation ranging from 700 to 1400 mm, depending on the elevation. The vegetation is dominated by mixed forests (600–1100 m), coniferous forests (1100–1800 m), sub-alpine Betula ermanii forests (1800–2000 m), or alpine tundra vegetation above 2000 m [55]. The CMA is one of the largest volcanic areas in East Asia, with volcanic tectonic geomorphology, water landscapes, glaciers, and periglacial landforms [56]. The east and southeast areas of the study area border North Korea and Russia. The study area includes seven counties and cities, including Changbai, Fusong, and Antu. The total area of the CMA is approximately 19,774 km^2^.

The topography is diverse, including valleys, basins, hills, and steep slopes. The overall trend of the terrain is centered on Baiyun Mountain and gradually reduced to the surrounding area, characterized by large height, deep cutting, and steep slopes. The highest point of the CMA is Baiyun Mountain, with an elevation of 2694 m. The lowest point is northwest of the Songhua River, with an elevation of 276 m.

The tectonic pattern of the area is dominated by NSS- and NNS-trending fault zones supplemented by SW-, SN- and NW-trending faults. The main faults include the Shulan-Yitong graben fault, the Dunhua-Mishan graben, and the Tumenjiang fault [57].

Extensive tectonic fragmentation, frequent volcanic activity, steep slopes, a dense drainage and road network, and extensive human activity, including North Korea’s nuclear testing and other factors, constitute a study area that is particularly prone to landslide phenomena.

## 3. Data Collection

**Figure 2:**
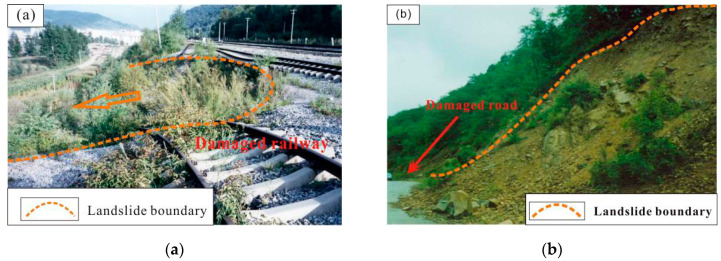
Field views of landslides considered in the area. (**a**) Wanlihe station landslide; (**b**) Qinxiang landslide.

Extensive field investigations and observations were identified and mapped in the CMA to produce a detailed and reliable landslide inventory map. In all, 116 landslides were identified and mapped in the study area by aerial photos supported by field investigation from 2012 to 2015. The landslides in the study area are characterized by two modes: slump-tensile rupture and creep-tensile rupture, both of them eventually leading to landslide instability. Of these landslides, 70% (81 landslides) were randomly selected for model training and the remaining 30% (35 landslides) were selected for validation. A series of field investigations were undertaken to identify the relationship between landslide occurrence and environmental factors. Figure 2 illustrates some typical landslides that destroyed railways and roads.

Thirteen layers of landslide conditioning factors, namely, altitude, slope gradient, slope aspect, lithology, distance to faults, distance to roads, distance to rivers, annual precipitation, land type, normalized difference vegetation index (NDVI), topographic wetness index (TWI), plan curvature, and profile curvature, were taken as independent, causal predictors for producing LSM. The selection of 13 predictors was based on the works of previous researchers, collection of data availability and the experience and knowledge about landslide activities in the study area [24,58]. The continuous predictors, such as altitude, slope gradient, slope aspect, distance to faults, distance to roads, distance to rivers, NDVI, TWI, plan curvature, and profile curvature, were classified according to natural break classes and the previous study, and the discrete predictors, including lithology, annual precipitation, and land type, were classified based on the existing classification. The spatial database for the study area is shown in Table 1.

The details about the data collection procedure and the preparation of the thematic layers are as follows:

The altitude map, slope gradient, slope aspect, plan curvature, and profile curvature were produced using the digital elevation model (DEM) with a grid size of 30 m × 30 m. In the present study area, the altitude ranges from 276 to 2694 m and is reclassified into five categories using natural break classes: 276–715 m, 715–924 m, 924–1162 m, 1162–1507 m, and 1507–2694 m. The slope gradient is reclassified into six classes, namely, 0–8°, 8–15°, 15–25°, 25–35°, 35–45°, and >45°. The slope aspect is reclassified into eight classes: North (−22.5–22.5°), Northeast (22.5–67.5°), East (67.5–112.5°), Southeast (112.5–157.5°), South (157.5–202.5°), Southwest (202.5–247.5°), West (247.5–302.5°), and Northwest (302.5–347.5). The plan curvature is reclassified into three classes: <-0.5, −0.5–0.5 and >0.5. The profile curvature is reclassified into three classes: <−0.5, −0.5–0.5 and >0.5.

Lithology, annual precipitation, land type, rivers, faults, and road feature maps were obtained from the China Geology survey. The distance to faults was classified into five classes: 0–500, 500–1000, 1000–1500, 1500–2000, and >2000 m. In the case of distance to rivers, there are five classes with 100 m intervals. For distance to roads, there are six classes: 0–500, 500–1000, 1000–1500, 1500–2000, and >2000 m.

The hydrological factor TWI was calculated using Equation (1). The TWI can be used as an estimate of spatial patterns for soil moisture, since topography controls the hydrological conditions of surface runoff and groundwater flow:

(1) $$TWI=\ln(A_{s}/\tan\beta ),$$

where $A_{s}$ is the specific catchment area and β is the slope gradient (in degrees). In the current study, TWI is divided into five classes: <9, 9–11, 11–14, 14–18, and >18.

The NDVI is a standardized index for generating an image displaying greenness, and it was prepared using Landsat-7 images based on the following equation:

(2) $$NDVI=\frac{N\mathrm{IR}-RED}{NIR+RED},$$

where NIR(band 4) and RED(band 3) are the infrared and red bands of the electromagnetic spectrum, respectively. This index outputs values between −1.0 and 1.0, mostly representing greenness, where any negative values are mainly generated from clouds, water, and snow, and values near zero are mainly generated from rock and bare soil [1]. In this study, the NDVI map was divided into five classes: <0.1, 0.1–0.3, 0.3–0.5, 0.5–0.7, and >0.7.

## 4. Methodology

### 4.1. The Rough Set Model

RS theory was proposed by Pawlak as a mathematical framework for approximate reasoning that considers uncertainty and vagueness in decision-making processes [59,60]. A rough set is used to modify the index weight of factor layers, which can make the distribution more reasonable. The operation steps are as follows:

Formally, a quaternion S=(U,A,V,f) is an information system, where $U=\{x_{1},x_{1},\cdots ,x_{m}\}$ is the non-empty finite set of objects called the universe;A is the non-empty finite set of attributes; $V=\underset{\alpha \in A}{U}V_{a}$, where $V_{a}$ is the range of attributes; and f:U×A→V is called an information function such that $f(x,a)\in V_{a}$ for ∀a∈A,∀x∈U, where A=C∪D and C∩D=Φ; C denotes the condition attribute set; and D is the decision attribute set. The knowledge expression system, with both condition and decision attributes, is a decision table.

The significance of different condition attribute sets for the decision attribute set is different, and some attributes are redundant. Rough set theory, by mining the potential relationship between the condition and the decision attribute sets in the knowledge system, obtains the weight values of different condition attributes. In decision-making systems, the importance $sig(C_{i})$ of the conditional attribute $C_{i}$ can be calculated as:

(3) $$sig(C_{i})=\frac{\gamma_{C}(D)-\gamma_{C-C_{i}}(D)}{\gamma_{C}(D)}=1-\frac{\gamma_{C-C_{i}}(D)}{\gamma_{C}(D)},$$

where $\gamma_{C}(D)$ reflects the dependency degree of the decision attribute set D on conditional set C, so $0\leq sig(C_{i})\leq 1$, $sig(C_{i})=0$ means that D does not depend on $C_{i}$; $0<sig(C_{i})<1$ means that D partly depends on $C_{i}$; $sig(C_{i})=1$ means that D totally depends on $C_{i}$, and the dependency $\gamma_{C}(D)$ is calculated using Equation (4). If $C_{i}$ is deleted from the conditional attribute set C, the dependency $\gamma_{C-C_{i}}(D)$ of the decision attribute set D to set $C-C_{i}$ is calculated using Equation (5):

(4) $$\gamma_{C}(D)=\frac{1}{\left|U\right|}\sum_{i=1}^{m}\left|\gamma_{C}(D_{i})\right|,$$

(5) $$\gamma_{C-C_{i}}(D)=\frac{1}{\left|U\right|}\sum_{i=1}^{m}\left|\gamma_{C-C_{i}}(D)\right|,$$

where |U| is the number of samples in set U.

Then, the index weight vector $\left[\alpha_{1},\alpha_{2},\cdots ,\alpha_{i},\cdots ,\alpha_{n}\right]$ can be obtained using Equation (6)”

(6) $$\alpha_{i}=\frac{sig(C_{i})}{\sum_{i=1}^{n}sig(C_{i})}(i=1,2,\cdots ,n).$$

### 4.2. The Analytic Hierarchy Process

The analytic hierarchy process (AHP), as a multi-criteria decision analysis method, was proposed by Saaty [61], and have been widely used in LSM [23,24]. The weights can be derived by taking the principal eigenvector of a square reciprocal matrix of pairwise comparisons between the criteria. The pairwise comparison of the 9-point rating scale is shown in Table 2. This approach can be described in four steps as follows [51,62,63]:

Step 1: Establish the hierarchical tree model for landslide susceptibility mapping;

Step 2: Build the judgement matrix based on pairwise comparison;

Step 3: Calculate the weights or the level of influence for each element based on the minimum of squares, the logarithmic minimum of squares, the special vector, or approximation methods;

Step 4: Check the consistency of the weights, called the consistency ratio (CR). The CR must be equal to or less than 10%; otherwise, the pairwise comparison values have to be recalculated.

### 4.3. The Entropy Weight Method

The entropy method is an objective method for calculating the weight of evaluation predictors based on measured values [19,40,64,65,66]. The operation steps are as follows:

Step 1: Establish matrix $X={\left(x_{ij}\right)}_{m\times n}\;(i=1,2,\cdots ,m;j=1,2,\cdots ,n)$ of the original evaluation data according to the evaluation objects and indicators, where m is the number of evaluation objects, and n is the number of evaluation indicators.

Step 2: Normalize matrix X.

For the cost type, the larger the better:

(7) $$y_{i}=\frac{x_{i}-x_{i(\min)}}{x_{i(max)}-x_{i(\min)}}.$$

For the efficiency type, the smaller the better:

(8) $$y_{i}=\frac{x_{i(\max)}-x_{i}}{x_{i(max)}-x_{i(\min)}}.$$

The standardization process yields the standard-grade matrix $Y={\left(y_{ij}\right)}_{m\times n}$.

Step 3: Calculate the entropy value $H_{i}$:

(9) $$H_{j}=-\frac{1}{LN(m)}\sum_{i=1}^{m}I_{ij}LN(I_{ij}),$$

where $I_{ij}=\frac{y_{ij}}{\sum_{i=1}^{m}y_{ij}}$, if $I_{ij}=0$, $LN(I_{ij})=0$.

Step 4: Calculate the weight of each indicator based on the entropy values:

(10) $$\omega_{j}=\frac{1-H_{j}}{n-\sum_{j=1}^{n}H_{j}}.$$

### 4.4. The Information Content Model

The information content model (ICM), as a statistical analysis method that has been used with good results for landslide susceptibility assessment [67], was introduced by C.E Shannon and derived from information theory. The ICM is used to calculate the effects of various engineering geological environments on landslides. The calculation procedure is as follows:

Step 1: Calculate the information content $I(X_{i},H)$ for each factor $X_{i}$ that influences landslide occurrence:

(11) $$I(X_{i},H)=\ln\frac{P(X_{i},H)}{P(X_{i})},$$

where $P(X_{i},H)$ is the probability of occurrence of $X_{i}$ in the landslide area and $P(X_{i})$ is the probability of occurrence of $X_{i}$ in the study area;

(12) $$I(x_{i},H)=\ln\frac{N_{i}/N}{S_{i}/S},$$

where N is the number of landslides in the study area, S is the total number of pixels in the study area, $N_{i}$ is the number of pixels for factor $X_{i}$ in the landslide area, and $S_{i}$ is the number of pixels for factor $X_{i}$ in the study area.

Step 2: Calculate the total information content for each factor $X_{i}$:

(13) $$I_{i}=\sum_{i=1}^{n}I(x_{i},H)=\sum_{i=1}^{n}\ln\frac{N_{i}/N}{S_{i}/S},$$

where $I_{i}$ is the total information content for factor $X_{i}$ and n is the total number of predictors. The greater the value, the more likely the landslide will occur.

### 4.5. The Landslide Susceptibility Assessment

Based on the ICM and combining with AHP, RS, and EWM, the information weight for each factor can be obtained using the following formula:

(14) $$I_{iw}=\sum_{i=1}^{n}I(x_{i},H)=\sum_{i=1}^{n}(W_{i}\times \ln\frac{N_{i}/N}{S_{i}/S}),$$

where $W_{i}$ are the weights for landslide conditioning predictors calculated by the AHP, RS, and EWM, and $I_{iw}$ is the comprehensive index of the landslide sensitivity.

### 4.6. Performance Evaluation

To assess the performance and measure the spatial consistency of the four models, the Cohen’s kappa index, a different measure of the reliability of a classification model, was used [68,69,70]. The Cohen’s kappa index is obtained as:

(15) $$k=\frac{P_{c}-P_{E}}{1-P_{E}},$$

where $P_{c}=\frac{TP+TN}{TP+TN+FP+FN}$ is the observed agreements and $P_{E}=\frac{\left(TP+FN)\times (TP+FP)+(FP+TN)\times (FN+TN\right)}{\sqrt{TP+TN+FP+FN}}$ is the expected agreements. Of these, FP, FN, TP, and TN are the number of false positive, false negative, true positive, and true negative, respectively.

In our case, a *k* value close to 0 means that the agreement is no better than chance, whereas a *k* value close to 1 indicates a perfect agreement.

## 5. Results

### 5.1. LSM using the ICM

**Figure 3:**
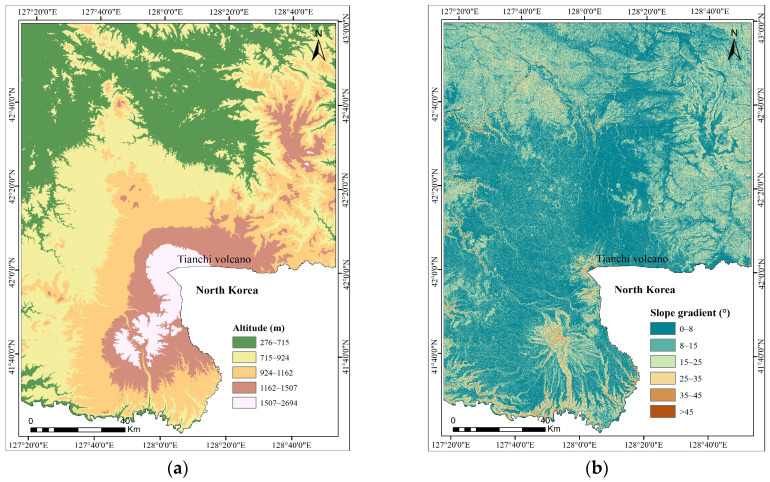

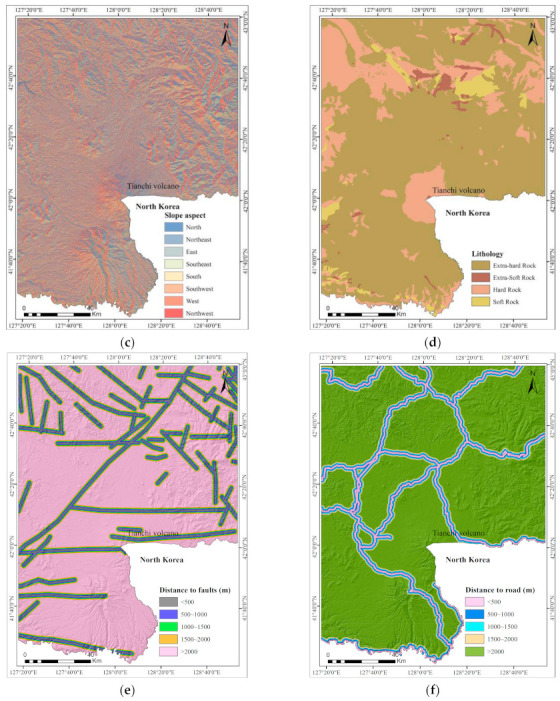

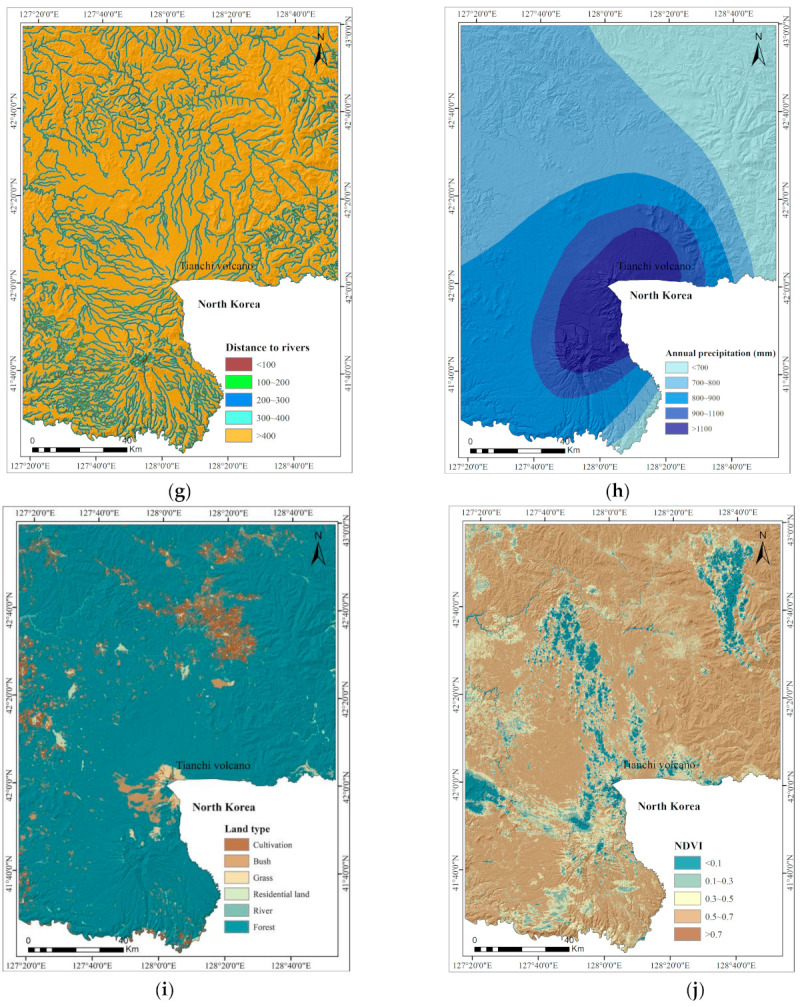

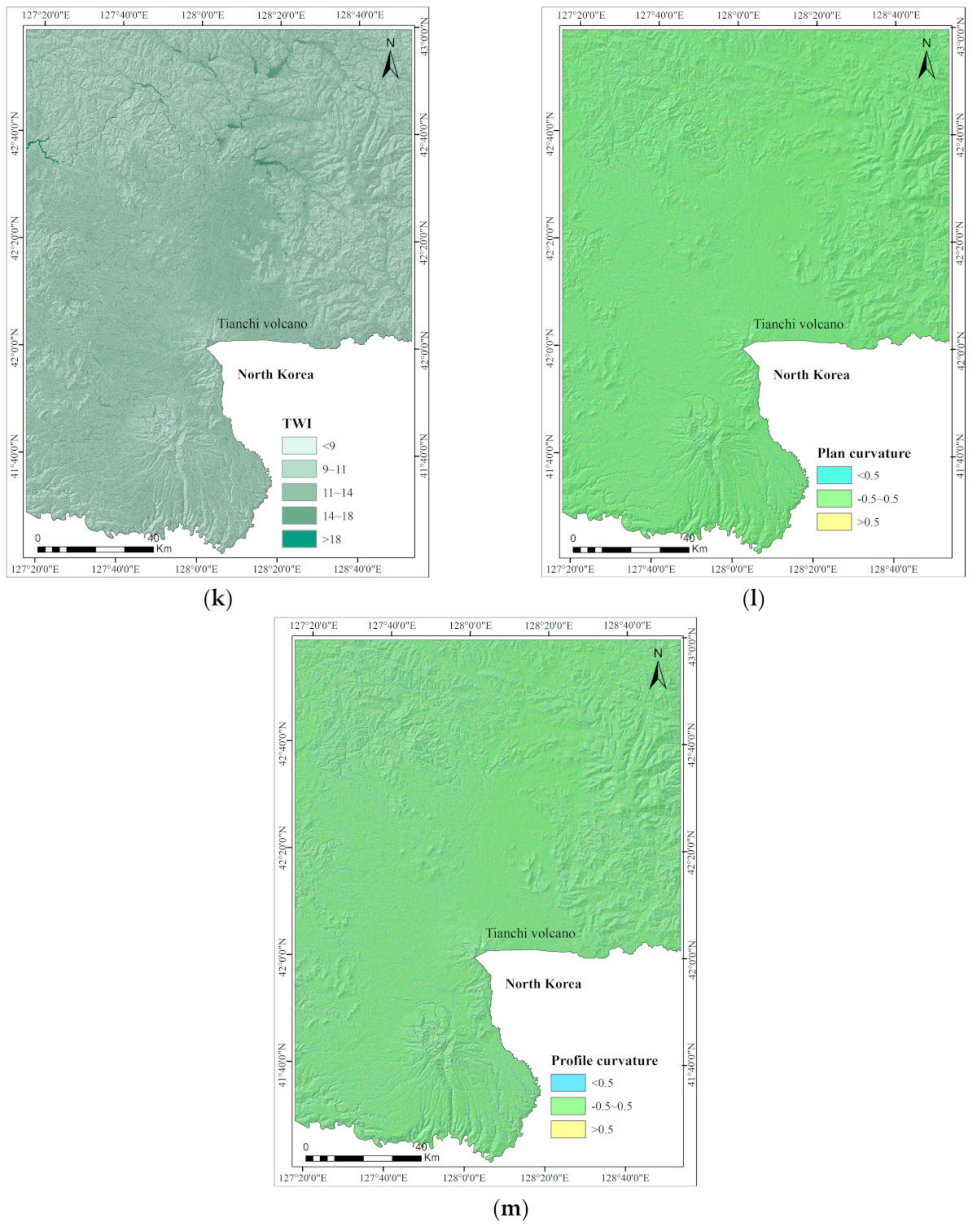
The study area’s influencing factor maps: (**a**) altitude; (**b**) slope gradient; (**c**) slope aspect; (**d**) lithology; (**e**) distance to faults; (**f**) distance to roads; (**g**) distance to rivers; (**h**) annual precipitation; (**i**) land type; (**j**) normalized difference vegetation index (NDVI); (**k**) topographic wetness index (TWI); (**l**) plan curvature; (**m**) profile curvature.

The information content (IC) for causative predictors was calculated with Equations (11), (12), and (13), and the results are listed in Table 3. The final thirteen-factor landslide susceptibility map obtained by the ICM is shown in Figure 3.

It is clear that the landslide occurrence increases with the slope gradient. The slope gradient class >45° has the highest IC value, and the lowest IC value is −0.76 for slope class 0–8°.

In the case of slope aspect, the IC value is positive from south to east, with the maximum value (0.89) at southeast-facing slopes followed by south-facing (0.36) slopes.

In terms of altitude, the IC values indicate they are positive for the ranges of 276–715 and 1507–2694, with the highest value for altitudes between 276–715 m.

For the lithology groups, Hard rock class is associated with a higher IC value, whereas the Extra-hard rock determines a lower IC value.

The plan and profile curvature IC values are negative only for the range of −0.5–0.5 and at −0.24 and −0.46, respectively. The concave (>0.5) and convex (<−0.5) slopes are positive for landslide susceptibility in the study area.

As for land types, Cultivation and River are more susceptible to landslides.

The relation between TWI landslide probabilities showed that >18 has the highest value of IC, and class 0.5–0.7 has the lowest NDVI value.

In terms of distance to faults, the highest and lowest IC values are located in the intervals of 1000–1500 m and >2000 m, respectively.

In the case of distance to roads, the interval <500 m has the highest IC value, which means that the landslide susceptibility is higher in this area.

The river incision can cause instability of slopes by changing groundwater level and toe erosion. Generally, with the increase of the distance to rivers the IC values decrease, and the IC value is positive only for the class >400 m.

In the case of annual precipitation, the landslides are mainly distributed within 700–800 mm and >1000 mm, and their IC values are all 0.33.

**Figure 4:**
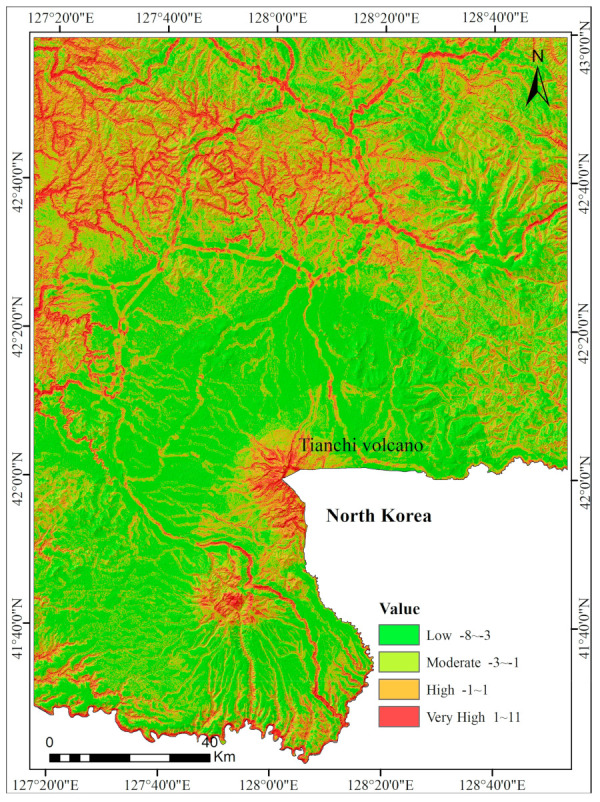
The landslide susceptibility map for the CMA extracted with the ICM.

The final ICM method landslide susceptibility map is shown in Figure 4.

### 5.2. LSM Using the RS-ICM Method

**Figure 5:**
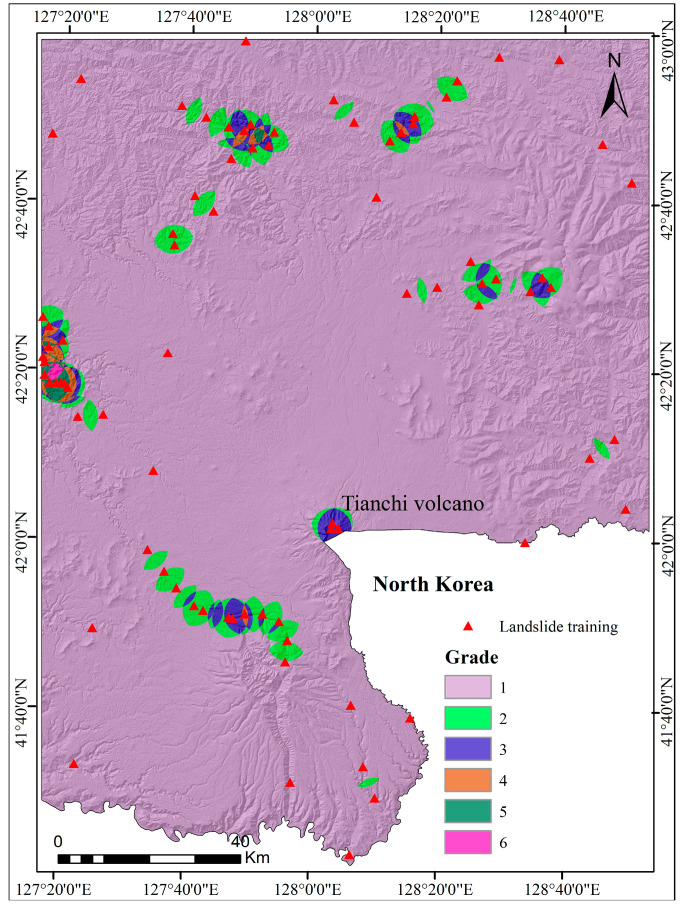
Landslide density maps based on landslide distribution.

**Figure 6:**
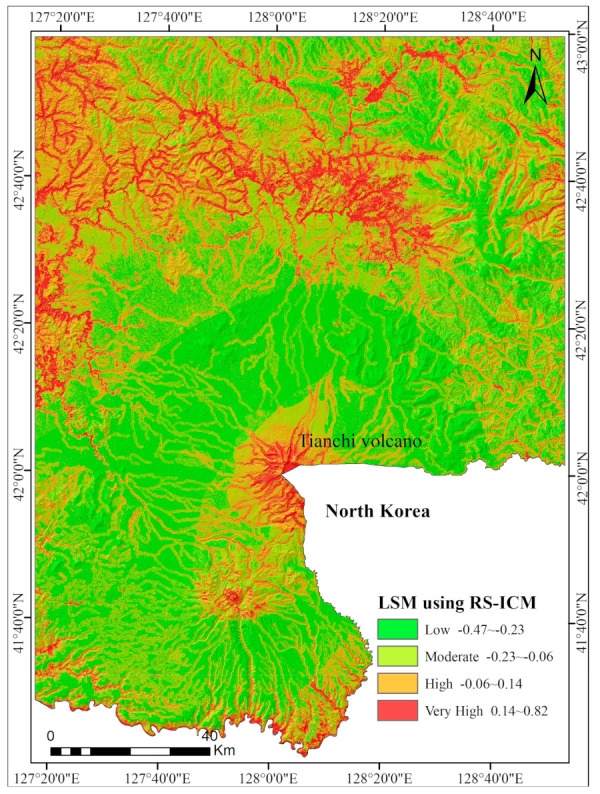
The landslide susceptibility map of the CMA extracted with the RS-ICM method.

The following predictors were selected as indices for LSM: slope gradient, slope aspect, lithology, distance to faults, distance to roads, distance to rivers, annual precipitation, land type, NDVI, TWI, plan curvature, and profile curvature. The 13 causative predictors were classified into grades 1, 2, 3, 4, 5, 6, 7, 8, and 9 according to landslide density as shown in Table 4. In ArcGIS software (version 10.2, Esri Co. Ltd., California, CA, USA), the value of all evaluation factor layers is extracted into landslides, and the results are used as condition attributes. We generated landslide density maps (Figure 5) based on the landslide distribution. The landslides were divided into six levels, 1, 2, 3, 4, 5, and 6, using natural break classification according to the landslide density, and the results were used as decision attributes. The initial decision table, with 85 rows and 8 columns, was established by defining the density of landslides as the decision attribute set. The weights of 13 predictors were calculated using the rough set method, as shown in Table 5. The final landslide RS-ICM susceptibility map is shown in Figure 6.

### 5.3. LSM Using the AHP-ICM Method

**Figure 7:**
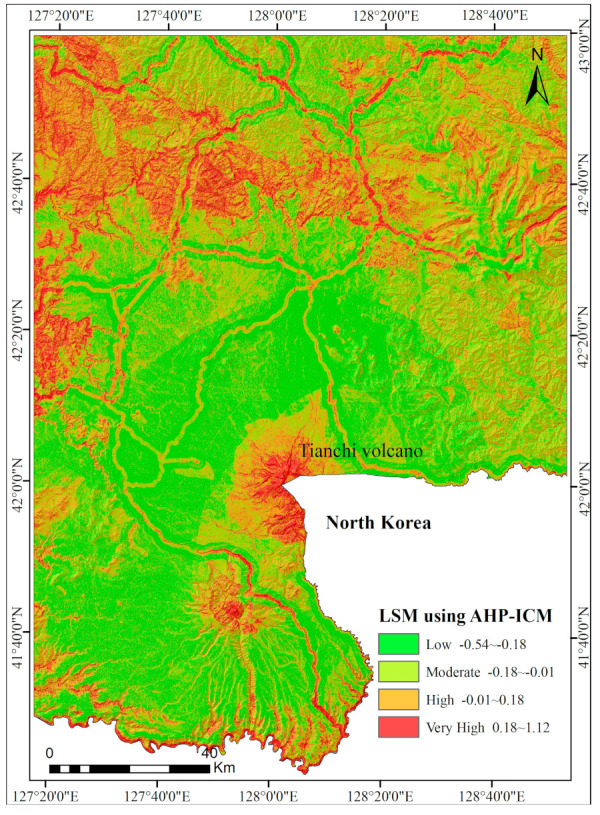
The landslide susceptibility map for the CMA extracted with the AHP-ICM method.

In the study area, the 13 layers of landslide conditioning predictors were compared with each other to determine their relative importance using the analytic hierarchy process (AHP) mentioned above. The judgement matrix for these evaluation predictors is shown in Table 6. The CR = 0.0183, which is <0.1, indicates that the calculated weights are reasonable. The final landslide susceptibility map of the AHP-ICM method is shown in Figure 7.

### 5.4. LSM Using the EWM-ICM Method

**Figure 8:**
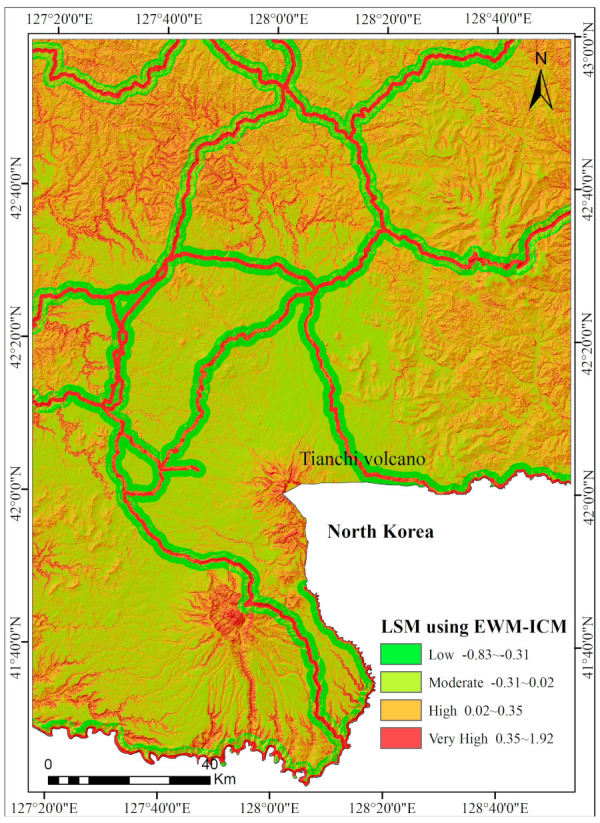
The CMA landslide susceptibility map extracted with the EWM-ICM method.

The weights of causative predictors based on EWM were calculated according to the principles of entropy weighted theory and are listed in Table 7. At first, the IC values were multiplied by the weights in Table 4 and all the weighted factor maps were then aggregated. Finally, the maps were reclassified to produce the EWM-ICM-generated LSM (Figure 8).

### 5.5. Validation

To determine the statistical reliability of the results, it is essential to perform validation of the four models. To perform this validation, the ROC curve was constructed, and the area under the ROC curve (AUC) was used for the quantitative comparison of the four models.

**Figure 9:**
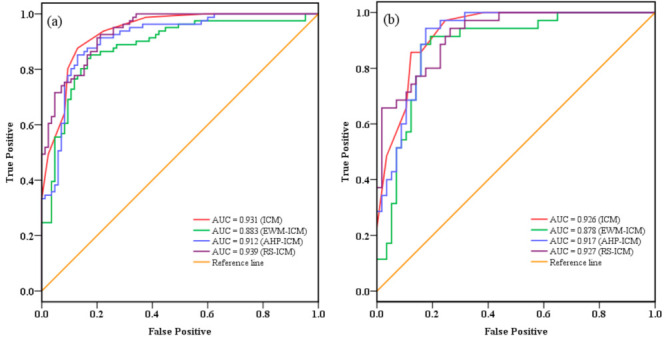
Receiver operating characteristic (ROC) curve evaluation of the four models: (**a**) success rate curve; (**b**) prediction rate curve.

The comparison results are shown in Figure 9. The success rate (Figure 9a) comes from the training dataset (70%, 81 landslides), and the prediction rate (Figure 9b) comes from the validation dataset (30%, 35 landslides). As shown in Figure 9, the ICM, RS-ICM, AHP-ICM, and EWM-ICM success rates were 0.931, 0.939, 0.912, and 0.883, and their prediction accuracy rates were 0.926, 0.927, 0.917, and 0.878, respectively. The Cohen’s kappa indexes were 0.721, 0.743, 0.720, and 0.663 for the ICM, RS-ICM, AHP-ICM, and EWM-ICM. It indicates a substantial agreement between the observed and the predicted values for all four models.

The susceptibility maps were classified as low, moderate, high, and very high based on a natural break approach. Table 8 displays the total area and ratios of low, moderate, high and very high susceptibility for the four models. The ICM, RS-ICM, and AHP-ICM methods with better LSM performance in the CMA have approximately the same result in area ratio of different landslide susceptibility classes, and they have relatively large differences compared to EWM-ICM.

## 6. Discussion

### 6.1. Spatial Consistency of the Four Models

The Cohen’s kappa indexes were calculated to measure the spatial consistency of the four models. The results of their spatial consistency are shown in Table 9, which indicates a substantial agreement between the ICM and AHP-ICM, and a moderate agreement between the RS-ICM and AHP-ICM and between the ICM and RS-ICM. For the RS-ICM and EWM-ICM, the Cohen’s kappa index was estimated to be equal to 0.140, characterizing with slight agreement. It should be noted that the Cohen’s kappa indexes between EWM-ICM, which has the lowest performance for LSM in the CMA, and the other three models are very small, which indicates that they have relatively large differences in spatial consistency compared to EWM-ICM, and the ICM, RS-ICM, and AHP-ICM methods yield similar results.

### 6.2. Predisposing Factors Analysis of Information

The ICM is a simple and effective tool in landslide susceptibility assessment, and the information values represent the contribution ratio of different predictor classes to landslide occurrence.

With the increase of slope gradient, the landslides increase. The reason for this is that the slope gradient not only affects the stress distribution inside the slope masses but also affects weathering layer depth and slope surface runoff [58]. However, the landslides are mainly concentrated in the altitude range of 276–715, which is due to the fact that the catchment areas are mainly concentrated in this range.

It should be noted that landslides primarily developed within the hard rock lithology group rather than soft rock. The hard rock group is mainly composed of basalt and trachyte, which are prone to rock fall, and the landslides in the study area are characterized by two modes: slump-tensile rupture and creep-tensile rupture, which are related to the development of rock fall. The landslides develop in the weak layer of the high-steep slope of the hard rock lithology group. Once the rock fall occurs at the front edge of the slope, the trailing edge will break through the weak layer along the weak edge and gradually form a connecting slip surface, forming a stepped rock fall-landslide, with conditions that are consistent with the occurrence conditions of landslide disasters.

In the case of distance to faults, distance to roads, and distance to rivers, there was a decreasing tendency with the increase in distance. The reasons for this phenomenon can be summed up as follows: (a) With strong weathering and a well-developed rock structure plane in a fault zone, it provides favorable conditions for a landslide occurrence; (b) Roads increase stress and strain on the back of the slope, resulting in slope disturbance and failure; (c) The strength of degree of surface incision is directly related to the development of drainages. The closer to the rivers, the more severe erosion, and the more landslides.

For slope aspect, the slope aspects in the south direction (south, southeast, and southwest) were more prone to landslides. The reason for this is that these direction slopes are exposed to more sunlight or affected by the orientation of discontinuities controlling the landslides, which is the same as the conclusion proposed by Du [58].

Annual precipitation is one of the major initiating factors of landslides [71]. It is accepted that with the increase of rainfall, the probability of landslide occurrence increases, but the results show that the IC values in the intervals of 800–900 and 900–1000 are negative, mainly because the two intervals are distributed in the Extra-hard rock group that is not prone to landslides.

It is generally accepted that Land type plays a crucial role in the landslide distribution [36]. The landslides are primarily developed with Cultivation and Residential in the study area, which are the most dramatic locations of human activity.

### 6.3. Comparative Analysis of Three Knowledge-Driven Methods

The ICM, an objective evaluation method commonly applied for statistical analysis, is suitable for the evaluation of LSM [24]. Four landslide susceptibility maps were produced based on the ICM with three knowledge-driven methods to mitigate the social and economic losses induced by landslides in the CMA. In the comprehensive evaluation process, the key issue is to determine the weight of each predictor, which reflects the relative importance of each evaluation indicator. It should be noted that the LSM is completely dependent on the value of the weight when the evaluation object and evaluation indicators are determined. Hence, the reasonable choice of the weighting method directly affects the rationality and credibility of the landslide-prone partition evaluation results. In this study, three knowledge-driven methods, including subjective evaluation and objective evaluation, were used to produce landslide susceptibility maps. The RS theory is an effective tool in dealing with vagueness and uncertainty information. Peng et al. [60] mentioned that the RS theory is an attribute reduction tool to identify the significant environmental parameters of a landslide. Liu et al. [72] used RS theory to clarify the relationship between landslide and environmental factors in the Qinggan River of the Three Gorges area. The AHP is an expert-based evaluation method that is often applied in landslide susceptibility assessment and mapping [63]. However, it should be noted that the AHP has been criticized for its inability to adequately handle the ambiguity and imprecision associated with the conversion of linguistic labels attached to the ratio scale, to crisp numbers used in the comparison matrix [73]. The EWM is an objective weighting method, which determines the criteria weights by solving mathematical models without any consideration of the decision maker’s preferences. However, it is sometimes contrary to the actual situation, and it is difficult to give a clear explanation for the obtained results. According to the results in the success accuracy section, the RS-ICM (AUC = 0.939) had the best effect for LSM in the CMA, and the ICM (AUC = 0.931) with AHP-ICM (AUC = 0.912) performed better than EWM-ICM (AUC = 0.883).

### 6.4. Importance of Predictors

**Figure 10:**
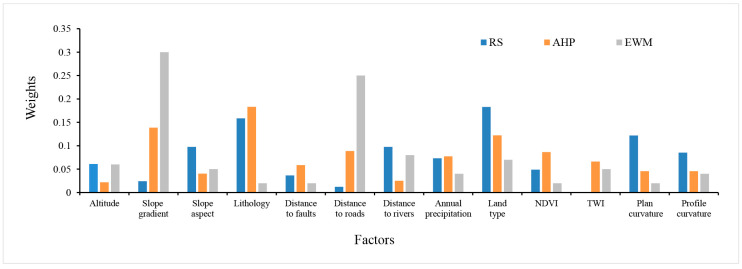
The columnar statistical graph of weights for the thirteen predictors of the three methods.

The weight assignment of landslide conditioning predictors is the basis of LSM. This paper focuses on the influence of different knowledge-driven methods for a series of parameters on the accuracy of LSM. Figure 10 displays the weights of the RS, AHP, and EWM methods. According to the results, lithology (0.1585), land type (0.1829), and plan curvature (0.1220) had the highest weights using RS. Slope gradient (0.1386), lithology (0.1832), and land type (0.1221) had the highest weights by AHP, and slope gradient (0.3) and distance to roads (0.25) had the highest weights using EWM. The results show that lithology and land type are crucial predictors for LSM in the CMA for RS-ICM (AUC = 0.939) and AHP-ICM (0.912), which have better performance than EWM-ICM (0.878). However, Chen et al. [52] found that slope gradient, altitude, and rainfall had the highest importance in landslide occurrence. Kawabata and Bandibas [74] mentioned that geology is the most important factor. Meinhardt, et al. [75] reported that slope gradient, lithology, and precipitation increase have higher importance in landslide occurrence. Pham, et al. [76] mentioned that distance to roads, slope gradient, elevation, and rainfall have higher importance on landslide occurrence, which is consistent with the results of Youssef et al. [77]. It can be found that slope gradient, lithology, distance to roads, and land type are the four most important predictors affecting the susceptibility of landslides in the CMA.

## 7. Conclusions

Landslides are one of the most frequent geomorphic hazards that often result in loss of property and human life in the CMA. In this research, 13 layers of landslide conditioning predictors, namely, altitude, slope gradient, slope aspect, lithology, distance to faults, distance to roads, distance to rivers, annual precipitation, land type, NDVI, TWI, plan curvature, and profile curvature, were taken as independent, causal predictors, and three knowledge-driven methods, that is, RS, AHP, and EWM, were applied to produce CMA landslide susceptibility maps and their performance was compared.

The influence of different knowledge-driven methods for a series of parameters on the accuracy of LSM was explored. The results demonstrate that the four models are good at predicting landslide susceptibility, and the RS-ICM had the best effect for LSM in the CMA; the next best were ICM and AHP-ICM, as their accuracies were slightly higher than that of EWM-ICM. In addition, the importance of different predictors in landslide occurrence was investigated. It was concluded that lithology and land type are crucial predictors for LSM in the CMA.

The four landslide susceptibility maps illustrated that the high-susceptibility areas were mainly composed of three parts: the region with a radius of 13,000 m around the Tianchi volcanic cone and mountain area in southwest Changbaishan Tianchi, the hard rock group area, and the surrounding area of roads. The outcome of this research is useful for general planned development activities and disaster management in the future and for engineers to reduce losses caused by existing and future landslides using prevention, mitigation, and avoidance.

## Figures and Tables

**Table 1:** Spatial database for the study area.

Data Layers	Data Type	Scale
Altitude	Grid	30 m × 30 m
Slope gradient	Grid	30 m × 30 m
Slope aspect	Grid	30 m × 30 m
Lithology	Polygon	1:100000
Annual precipitation	Polygon	1:100000
Land type	Polygon	1:100000
Distance to rivers	Polygon	1:100000
Distance to faults	Polygon	1:100000
Distance to roads	Polygon	1:100000
Plan curvature	Grid	30 m × 30 m
Profile curvature	Grid	30 m × 30 m
Topographic wetness index (TWI)	Grid	30 m × 30 m
Normalized difference vegetation index (NDVI)	Grid	30 m × 30 m

**Table 2:** Pair-wise comparison of 9-point rating scale.

Importance	Definition
1	Equal importance
3	Moderate prevalence of one over another
5	Strong or essential prevalence
7	Very strong or demonstrated prevalence
9	Extremely high prevalence
2, 4, 6, 8	Intermediate values

**Table 3:** Information content for causative predictors.

Factor	Class	Number of Landslides	Total Count	Information Content	Landslide density (one/km^2^)
Altitude/m	276–715	46	6,092,214	0.69	0.0084
	715–924	15	6,952,902	−0.57	0.0024
	924–1162	12	4,897,656	−0.44	0.0027
	1162–1507	4	2,570,941	−0.89	0.0017
	1507–2694	4	794,775	0.28	0.0056
Slope gradient/°	0–8	16	8,989,246	−0.76	0.0020
	8–15	23	6,698,970	−0.10	0.0038
	15–25	21	4,135,676	0.29	0.0056
	25–35	12	1,175,687	0.99	0.0113
	35–45	4	263,478	1.38	0.0169
	>45	5	45,431	3.37	0.1223
Lithology group	Extra-hard rock	45	15,716,967	−0.28	0.0032
	Hard rock	30	4,450,560	0.57	0.0075
	Soft rock	4	721,619	0.38	0.0062
	Extra-soft rock	2	401,503	0.27	0.0055
Distance to faults/m	<500	8	1,562,977	0.30	0.0057
	500–1000	6	1,590,516	−0.01	0.0042
	1000–1500	10	1,603,260	0.50	0.0069
	1500–2000	4	1,593,415	−0.41	0.0028
	>2000	53	14,958,243	−0.07	0.0039
Slope aspect	North	6	2,874,520	−0.60	0.0023
	Northeast	6	2,665,103	−0.52	0.0025
	East	6	2,713,061	−0.54	0.0025
	Southeast	8	2,311,122	−0.09	0.0038
	South	14	2,559,523	0.36	0.0061
	Southwest	24	2,584,545	0.89	0.0103
	West	12	2,931,061	0.07	0.0045
	Northwest	5	2,669,553	−0.71	0.0021
Distance to roads/m	<500	41	1,099,232	2.28	0.0414
	500–1000	2	982,690	−0.63	0.0023
	1000–1500	2	921,682	−0.56	0.0024
	1500–2000	1	882,297	−1.21	0.0013
	>2000	35	17,416,244	−0.64	0.0022
Distance to rivers/m	0–100	9	1,924,137	0.21	0.0052
	100–200	27	1,868,346	1.34	0.0161
	200–300	14	1,790,363	0.72	0.0087
	300–400	8	1,682,849	0.22	0.0053
	>400	23	14,042,793	−0.84	0.0018
Annual precipitation/mm	<700	18	4,524,190	0.05	0.0044
	700–800	41	7,781,530	0.33	0.0059
	800–900	9	5,299,708	−0.80	0.0019
	900–1100	3	1,846,085	−0.85	0.0018
	>1100	10	1,849,861	0.33	0.0059
Land type	Cultivation	13	929,169	1.30	0.0155
	Bush	2	662,587	−0.23	0.0034
	Grass	1	176,242	0.40	0.0063
	Residential land	1	236,892	0.10	0.0047
	River	3	192,095	1.41	0.0174
	Forest	61	19,105,160	−0.17	0.0035
NDVI	<0.1	7	1,306,736	0.34	0.0060
	0.1–0.3	11	1,534,455	0.63	0.0080
	0.3–0.5	22	3,657,943	0.46	0.0067
	0.5–0.7	40	14,605,211	−0.33	0.0030
	>0.7	1	187,856	0.34	0.0059
TWI	<9	43	7,275,471	0.44	0.0066
	9–11	23	9,193,638	−0.42	0.0028
	11–14	8	3,649,323	−0.55	0.0024
	14–18	5	964,716	0.31	0.0058
	>18	2	225,340	0.85	0.0099
Plan curvature	<0.5	18	3,382,177	0.34	0.0059
	−0.5–0.5	43	14,431,242	−0.24	0.0033
	>0.5	20	3,495,068	0.41	0.0064
Profile curvature	<0.5	24	4,378,283	0.37	0.0061
	−0.5–0.5	30	12,458,967	−0.46	0.0027
	>0.5	27	4,471,238	0.46	0.0067

**Table 4:** Evaluation class based on landslide density.

Landslide Density (one/km^2^)	Landslide Susceptibility Grade
0.001–0.002	1
0.002–0.003	2
0.003–0.004	3
0.004–0.005	4
0.005–0.006	5
0.006–0.007	6
0.007–0.008	7
0.008–0.009	8
>0.009	9

**Table 5:** Weights of 13 predictors using the rough set approach.

Predictors	X_1_	X_2_	X_3_	X_4_	X_5_	X_6_	X_7_	X_8_	X_9_	X_10_	X_11_	X_12_	X_13_
Weights	0.0610	0.0244	0.0976	0.1585	0.0366	0.0122	0.0976	0.0732	0.1829	0.0488	0	0.1220	0.0854

Notes: X_1_: Altitude; X_2_: Slope gradient; X_3_: Slope aspect; X_4_: Lithology; X_5_: Distance to faults; X_6_: Distance to roads; X_7_: Distance to rivers; X_8_: Annual precipitation; X_9_: Land type; X_10_: NDVI; X_11_: TWI; X_12_: Plan curvature; X_13_: Profile curvature.

**Table 6:** Pair-wise comparison matrix for influencing factor weights.

Heading	X_1_	X_2_	X_3_	X_4_	X_5_	X_6_	X_7_	X_8_	X_9_	X_10_	X_11_	X_12_	X_13_	Weights
**X_1_**	1	1/6	1/2	1/7	1/3	1/4	1	1/4	1/5	1/4	1/3	1/2	1/2	0.0218
**X_2_**	6	1	4	1/2	3	2	5	3	1	1	2	3	3	0.1386
**X_3_**	2	1/4	1	1/5	1	1/3	2	1/2	1/3	1/2	1/2	1	1	0.0405
**X_4_**	7	2	5	1	4	2	5	3	1	2	3	4	4	0.1832
**X_5_**	3	1/3	1	1/4	1	1/2	2	1/2	1/2	2	1	1	1	0.0586
**X_6_**	4	1/2	3	1/2	2	1	3	1	1	1	1	2	2	0.0889
**X_7_**	1	1/5	1/2	1/5	1/2	1/3	1	1/3	1/4	1/4	1/3	1/2	1/2	0.0252
**X_8_**	4	1/3	2	1/3	2	1	3	1	1/2	1	1	2	2	0.0773
**X_9_**	5	1	3	1	2	1	4	2	1	1	2	3	3	0.1221
**X_10_**	4	1	2	1/2	1/2	1	4	1	1	1	1	2	2	0.0864
**X_11_**	3	1/2	2	1/3	1	1	3	1	1/2	1	1	1	1	0.0661
**X_12_**	2	1/3	1	1/4	1	1/2	2	1/2	1/3	1/2	1	1	1	0.0457
**X_13_**	2	1/3	1	1/4	1	1/2	2	1/2	1/3	1/2	1	1	1	0.0457

**Table 7:** The weights of 13 predictors using the EWM approach.

Predictors	X_1_	X_2_	X_3_	X_4_	X_5_	X_6_	X_7_	X_8_	X_9_	X_10_	X_11_	X_12_	X_13_
**Weights**	0.06	0.30	0.05	0.02	0.02	0.25	0.08	0.04	0.07	0.02	0.05	0.02	0.04

**Table 8:** Distribution of area in different landslide susceptibility classes.

Model	Area (km^2^) and Ratio (%)	Susceptibility			
		Low	Moderate	High	Very High
ICM	Area	642.67	676.50	414.96	181.83
	Ratio	33.54	35.31	21.66	9.49
RS-ICM	Area	522.98	727.38	486.82	178.77
	Ratio	27.30	37.96	25.41	9.33
AHP-ICM	Area	515.62	697.16	505.96	197.22
	Ratio	26.91	36.39	26.41	10.29
EWM-ICM	Area	165.52	704.41	824.07	221.96
	Ratio	8.64	36.77	43.01	11.58

**Table 9:** Cohen’s kappa index between two models.

Models	ICM and RS-ICM	ICM and AHP-ICM	ICM and EWM-ICM	RS-ICM and AHP-ICM	RS-ICM and EWM-ICM	AHP-ICM and EWM-ICM
Cohen’s Kappa Index	0.595	0.698	0.325	0.484	0.140	0.286

## References

[1] Ahmed B. (2014). Landslide susceptibility mapping using multi-criteria evaluation techniques in Chittagong Metropolitan Area, Bangladesh. Landslides.

[2] Dagdelenler G., Nefeslioglu H.A., Gokceoglu C. (2015). Modification of seed cell sampling strategy for landslide susceptibility mapping: An application from the Eastern part of the Gallipoli Peninsula (Canakkale, Turkey). Bull. Eng. Geol. Environ..

[3] Chen W., Pourghasemi H.R., Kornejady A., Zhang N. (2017). Landslide spatial modeling: Introducing new ensembles of ANN, MaxEnt, and SVM machine learning techniques. Geoderma.

[4] Yeon Y.-K., Han J.-G., Ryu K.H. (2010). Landslide susceptibility mapping in Injae, Korea, using a decision tree. Eng. Geol..

[5] Reichenbach P., Rossi M., Malamud B.D., Mihir M., Guzzetti F. (2018). A review of statistically-based landslide susceptibility models. Earth-Sci. Rev..

[6] Abdulwahid W.M., Pradhan B. (2016). Landslide vulnerability and risk assessment for multi-hazard scenarios using airborne laser scanning data (LiDAR). Landslides.

[7] Cascini L. (2008). Applicability of landslide susceptibility and hazard zoning at different scales. Eng. Geol..

[8] Ercanoglu M., Temiz F.A. (2011). Application of logistic regression and fuzzy operators to landslide susceptibility assessment in Azdavay (Kastamonu, Turkey). Environ. Earth Sci..

[9] Akgun A. (2012). A comparison of landslide susceptibility maps produced by logistic regression, multi-criteria decision, and likelihood ratio methods: A case study at izmir, Turkey. Landslides.

[10] Bai S.-B., Wang J., Lü G.-N., Zhou P.-G., Hou S.-S., Xu S.-N. (2010). GIS-based logistic regression for landslide susceptibility mapping of the Zhongxian segment in the Three Gorges area, China. Geomorphology.

[11] Keefer D.K., Larsen M.C. (2007). Assessing Landslide Hazards. Science.

[12] Guzzetti F., Carrara A., Cardinali M., Reichenbach P. (1999). Landslide hazard evaluation: A review of current techniques and their application in a multi-scale study, Central Italy. Geomorphology.

[13] Park S., Choi C., Kim B., Kim J. (2013). Landslide susceptibility mapping using frequency ratio, analytic hierarchy process, logistic regression, and artificial neural network methods at the Inje area, Korea. Environ. Earth Sci..

[14] Zezere J.L., Pereira S., Melo R., Oliveira S.C., Garcia R.A.C. (2017). Mapping landslide susceptibility using data-driven methods. Sci. Total Environ..

[15] Choi J., Oh H.-J., Lee H.-J., Lee C., Lee S. (2012). Combining landslide susceptibility maps obtained from frequency ratio, logistic regression, and artificial neural network models using ASTER images and GIS. Eng. Geol..

[16] Kouli M., Loupasakis C., Soupios P., Rozos D., Vallianatos F. (2014). Landslide susceptibility mapping by comparing the WLC and WofE multi-criteria methods in the West Crete Island, Greece. Environ. Earth Sci..

[17] Corominas J., van Westen C., Frattini P., Cascini L., Malet J.P., Fotopoulou S., Catani F., Van Den Eeckhaut M., Mavrouli O., Agliardi F. (2014). Recommendations for the quantitative analysis of landslide risk. Bull. Eng. Geol. Environ..

[18] Duman T.Y., Çan T., Emre Ö., Keçer M., Doğan A., Ateş Ş., Durmaz S. (2005). Landslide inventory of northwestern Anatolia, Turkey. Eng. Geol..

[19] Kornejady A., Ownegh M., Bahremand A. (2017). Landslide susceptibility assessment using maximum entropy model with two different data sampling methods. Catena.

[20] Regmi A.D., Yoshida K., Pourghasemi H.R., DhitaL M.R., Pradhan B. (2014). Landslide susceptibility mapping along Bhalubang—Shiwapur area of mid-Western Nepal using frequency ratio and conditional probability models. J. Mt. Sci..

[21] Ozdemir A. (2015). Sinkhole Susceptibility Mapping Using a Frequency Ratio Method and GIS Technology Near Karapınar, Konya-Turkey. Procedia Earth Planet. Sci..

[22] Li L., Lan H., Guo C., Zhang Y., Li Q., Wu Y. (2016). A modified frequency ratio method for landslide susceptibility assessment. Landslides.

[23] Cao C., Wang Q., Chen J., Ruan Y., Zheng L., Song S., Niu C. (2016). Landslide Susceptibility Mapping in Vertical Distribution Law of Precipitation Area: Case of the Xulong Hydropower Station Reservoir, Southwestern China. Water.

[24] Wang F., Xu P., Wang C., Wang N., Jiang N. (2017). Application of a GIS-Based Slope Unit Method for Landslide Susceptibility Mapping along the Longzi River, Southeastern Tibetan Plateau, China. ISPRS Int. J. Geo-Inf..

[25] Devkota K.C., Regmi A.D., Pourghasemi H.R., Yoshida K., Pradhan B., Ryu I.C., Dhital M.R., Althuwaynee O.F. (2012). Landslide susceptibility mapping using certainty factor, index of entropy and logistic regression models in GIS and their comparison at Mugling–Narayanghat road section in Nepal Himalaya. Nat. Hazards.

[26] Zêzere J.L., Reis E., Garcia R., Oliveira S. (2004). Integration of spatial and temporal data for the definition of different landslide hazard scenarios in the area north of Lisbon (Portugal). Nat. Hazards Earth Syst. Sci. Discuss..

[27] Lee S. (2005). Application of logistic regression model and its validation for landslide susceptibility mapping using GIS and remote sensing data. Int. J. Remote Sens..

[28] Budimir M.E.A., Atkinson P.M., Lewis H.G. (2015). A systematic review of landslide probability mapping using logistic regression. Landslides.

[29] Elkadiri R., Sultan M., Youssef A.M., Elbayoumi T., Chase R., Bulkhi A.B., Al-Katheeri M.M. (2014). A Remote Sensing-Based Approach for Debris-Flow Susceptibility Assessment Using Artificial Neural Networks and Logistic Regression Modeling. IEEE J. Sel. Top. Appl. Earth Obs. Remote Sens..

[30] Greco R., Sorriso-Valvo M., Catalano E. (2007). Logistic Regression analysis in the evaluation of mass movements susceptibility: The Aspromonte case study, Calabria, Italy. Eng. Geol..

[31] Hong H., Pradhan B., Xu C., Tien Bui D. (2015). Spatial prediction of landslide hazard at the Yihuang area (China) using two-class kernel logistic regression, alternating decision tree and support vector machines. Catena.

[32] Lee S., Pradhan B. (2007). Landslide hazard mapping at Selangor, Malaysia using frequency ratio and logistic regression models. Landslides.

[33] Lee S., Ryu J.-H., Kim I.-S. (2007). Landslide susceptibility analysis and its verification using likelihood ratio, logistic regression, and artificial neural network models: Case study of Youngin, Korea. Landslides.

[34] Guzzetti F., Galli M., Reichenbach P., Ardizzone F., Cardinali M.J.N.H. (2006). Landslide hazard assessment in the Collazzone area, Umbria, Central Italy. Nat. Hazards Earth Syst. Sci..

[35] Lee C.-T., Huang C.-C., Lee J.-F., Pan K.-L., Lin M.-L., Dong J.-J.J.E.G. (2008). Statistical approach to earthquake-induced landslide susceptibility. Eng. Geol..

[36] Hong H., Pourghasemi H.R., Pourtaghi Z.S. (2016). Landslide susceptibility assessment in Lianhua County (China): A comparison between a random forest data mining technique and bivariate and multivariate statistical models. Geomorphology.

[37] Pourghasemi H.R., Kerle N. (2016). Random forests and evidential belief function-based landslide susceptibility assessment in Western Mazandaran Province, Iran. Environ. Earth Sci..

[38] Youssef A.M., Pourghasemi H.R., Pourtaghi Z.S., Al-Katheeri M.M. (2015). Landslide susceptibility mapping using random forest, boosted regression tree, classification and regression tree, and general linear models and comparison of their performance at Wadi Tayyah Basin, Asir Region, Saudi Arabia. Landslides.

[39] Imaizumi F., Hattanji T., Hayakawa Y.S. (2010). Channel initiation by surface and subsurface flows in a steep catchment of the Akaishi Mountains, Japan. Geomorphology.

[40] Cao C., Xu P., Chen J., Zheng L., Niu C. (2016). Hazard Assessment of Debris-Flow along the Baicha River in Heshigten Banner, Inner Mongolia, China. Int. J. Environ. Res. Public Health.

[41] Oh H.-J., Lee S. (2017). Shallow Landslide Susceptibility Modeling Using the Data Mining Models Artificial Neural Network and Boosted Tree. Appl. Sci..

[42] Lee S., Lee M.-J., Jung H.-S. (2017). Data Mining Approaches for Landslide Susceptibility Mapping in Umyeonsan, Seoul, South Korea. Appl. Sci..

[43] Yilmaz I. (2009). Landslide susceptibility mapping using frequency ratio, logistic regression, artificial neural networks and their comparison: A case study from Kat landslides (Tokat—Turkey). Comput. Geosci..

[44] Anagnostopoulos G.G., Fatichi S., Burlando P. (2015). An advanced process-based distributed model for the investigation of rainfall-induced landslides: The effect of process representation and boundary conditions. Water Resour. Res..

[45] Borga M., Dalla Fontana G., Cazorzi F. (2002). Analysis of topographic and climatic control on rainfall-triggered shallow landsliding using a quasi-dynamic wetness index. J. Hydrol..

[46] Ma Z., Qin S., Chen J., Lv J., Chen J., Zhao X. (2017). A probabilistic method for evaluating wedge stability based on blind data theory. Bull. Eng. Geol. Environ..

[47] Peres D.J., Cancelliere A. (2016). Estimating return period of landslide triggering by Monte Carlo simulation. J. Hydrol..

[48] Peres D.J., Cancelliere A. (2018). Modeling impacts of climate change on return period of landslide triggering. J. Hydrol..

[49] Salciarini D., Godt J.W., Savage W.Z., Baum R.L., Conversini P. (2008). Modeling landslide recurrence in Seattle, Washington, USA. Eng. Geol..

[50] Huang F., Yin K., Huang J., Gui L., Wang P. (2017). Landslide susceptibility mapping based on self-organizing-map network and extreme learning machine. Eng. Geol..

[51] Hung L.Q., van N.T.H., Duc D.M., Ha L.T.C., van Son P., Khanh N.H., Binh L.T. (2015). Landslide susceptibility mapping by combining the analytical hierarchy process and weighted linear combination methods: A case study in the upper Lo River catchment (Vietnam). Landslides.

[52] Chen W., Pourghasemi H.R., Naghibi S.A. (2017). A comparative study of landslide susceptibility maps produced using support vector machine with different kernel functions and entropy data mining models in China. Bull. Eng. Geol. Environ..

[53] Ba Q., Chen Y., Deng S., Yang J., Li H. (2018). A comparison of slope units and grid cells as mapping units for landslide susceptibility assessment. Earth Sci. Inform..

[54] Wang Y., Li C., Wei H., Shan X. (2003). Late Pliocene–recent tectonic setting for the Tianchi volcanic zone, Changbai Mountains, northeast China. J. Asian Earth Sci..

[55] Gao C., Knorr K.-H., Yu Z., He J., Zhang S., Lu X., Wang G. (2016). Black carbon deposition and storage in peat soils of the Changbai Mountain, China. Geoderma.

[56] Wang Y., Guan L., Piao Z., Wang Z., Kong Y. (2017). Monitoring wildlife crossing structures along highways in Changbai Mountain, China. Transp. Res. Part D Transp. Environ..

[57] Guo Z., Liu J., Han J., He H., Dau G., You H. (2006). Effect of gas emissions from Tianchi volcano (NE China) on environment and its potential volcanic hazards. Sci. China Ser. D.

[58] Du G., Zhang Y., Iqbal J., Yang Z., Yao X. (2017). Landslide susceptibility mapping using an integrated model of information value method and logistic regression in the Bailongjiang watershed, Gansu Province, China. J. Mt. Sci..

[59] Pawlak Z.A. (1982). Rough sets. Int. J. Comput. Inf. Sci..

[60] Peng L., Niu R., Huang B., Wu X., Zhao Y., Ye R. (2014). Landslide susceptibility mapping based on rough set theory and support vector machines: A case of the Three Gorges area, China. Geomorphology.

[61] Saaty T.L. (1978). Modeling unstructured decision problems-the theory of analytical hierarchies. Math. Comput. Simul..

[62] Alami Merrouni A., Elwali Elalaoui F., Mezrhab A., Mezrhab A., Ghennioui A. (2018). Large scale PV sites selection by combining GIS and Analytical Hierarchy Process. Case study: Eastern Morocco. Renew. Energy.

[63] Sangchini E.K., Emami S.N., Tahmasebipour N., Pourghasemi H.R., Naghibi S.A., Arami S.A., Pradhan B. (2016). Assessment and comparison of combined bivariate and AHP models with logistic regression for landslide susceptibility mapping in the Chaharmahal-e-Bakhtiari Province, Iran. Arab. J. Geosci..

[64] Al-Abadi A.M., Pourghasemi H.R., Shahid S., Ghalib H.B. (2016). Spatial Mapping of Groundwater Potential Using Entropy Weighted Linear Aggregate Novel Approach and GIS. Arab. J. Sci. Eng..

[65] Wang Q., Li W., Yan S., Wu Y., Pei Y. (2016). GIS based frequency ratio and index of entropy models to landslide susceptibility mapping (Daguan, China). Environ. Earth Sci..

[66] Zhao H., Yao L., Mei G., Liu T., Ning Y. (2017). A Fuzzy Comprehensive Evaluation Method Based on AHP and Entropy for a Landslide Susceptibility Map. Entropy.

[67] Xu W., Yu W., Jing S., Zhang G., Huang J. (2013). Debris flow susceptibility assessment by GIS and information value model in a large-scale region, Sichuan Province (China). Nat. Hazards.

[68] Cohen J. (1960). A coefficient of agreement for nominal scales. Educ. Psychol. Meas..

[69] Tsangaratos P., Ilia I. (2015). Landslide susceptibility mapping using a modified decision tree classifier in the Xanthi Perfection, Greece. Landslides.

[70] Rossi M., Guzzetti F., Reichenbach P., Mondini A.C., Peruccacci S. (2010). Optimal landslide susceptibility zonation based on multiple forecasts. Geomorphology.

[71] Ko F.W.Y., Lo F.L.C. (2016). Rainfall-based landslide susceptibility analysis for natural terrain in Hong Kong—A direct stock-taking approach. Eng. Geol..

[72] Liu J.P., Zeng Z.P., Liu H.Q., Wang H.B. (2011). A rough set approach to analyze factors affecting landslide incidence. Comput. Geosci..

[73] Feizizadeh B., Blaschke T. (2013). GIS-multicriteria decision analysis for landslide susceptibility mapping: Comparing three methods for the Urmia lake basin, Iran. Nat. Hazards.

[74] Kawabata D., Bandibas J. (2009). Landslide susceptibility mapping using geological data, a DEM from ASTER images and an Artificial Neural Network (ANN). Geomorphology.

[75] Meinhardt M., Fink M., Tünschel H. (2015). Landslide susceptibility analysis in central Vietnam based on an incomplete landslide inventory: Comparison of a new method to calculate weighting factors by means of bivariate statistics. Geomorphology.

[76] Pham B.T., Tien Bui D., Prakash I., Nguyen L.H., Dholakia M.B. (2017). A comparative study of sequential minimal optimization-based support vector machines, vote feature intervals, and logistic regression in landslide susceptibility assessment using GIS. Environ. Earth Sci..

[77] Youssef A.M., Pourghasemi H.R., El-Haddad B.A., Dhahry B.K. (2015). Landslide susceptibility maps using different probabilistic and bivariate statistical models and comparison of their performance at Wadi Itwad Basin, Asir Region, Saudi Arabia. Bull. Eng. Geol. Environ..

